# COVID-19 Vaccine: A Possible Trigger for Pyoderma Gangrenosum

**DOI:** 10.7759/cureus.25295

**Published:** 2022-05-24

**Authors:** Ahmed B Mohd, Omar B Mohd, Reem A Ghannam, Mohammad H Al-thnaibat

**Affiliations:** 1 Medicine, Hashemite University, Zarqa, JOR; 2 Basic Sciences, Faculty of Medicine, Hashemite University, Zarqa, JOR; 3 Clinical Sciences, Faculty of Medicine, Hashemite University, Zarqa, JOR; 4 Nephrology, Faculty of Medicine, Hashemite University, Zarqa, JOR

**Keywords:** inflammatory mediators, nonhealing ulcer, covid-19 vaccine, sinopharm bbibp, pyoderma gangrenosum

## Abstract

Pyoderma gangrenosum is an uncommon ulcerative auto-inflammatory dermatosis. Numerous studies suggest cutaneous side effects of the COVID-19 vaccine. Pyoderma gangrenosum has been reported as one of the rare side effects of the COVID-19 vaccine.

In this report, a 36-year-old male was admitted to a hospital due to a progression of pyoderma gangrenosum on the lateral aspect of his upper arm which had developed eight months ago, following the first dose of Sinopharm BBIBP COVID-19 vaccine. The reported symptoms included headache, blurred vision, palpitation, fatigue on exertion, documented fever, chills and productive cough with yellow sputum, possibly due to the inflammatory effect of pyoderma gangrenosum. In the past, the patient's face had several abnormal skin lesions similar to the newly developed lesion. In addition, the newly developed lesion did not regress despite using medication.

COVID-19 vaccinations could potentially trigger pyoderma gangrenosum, especially in patients with a past medical history of similar lesions in different body parts. Therefore, we recommend inquiring about the past medical history of pyoderma gangrenosum or abnormal skin lesions prior to vaccination.

## Introduction

Coronavirus disease 2019 (COVID-19) is a multisystem disease caused by severe acute respiratory syndrome coronavirus 2 (SARS-CoV-2), which primarily causes respiratory symptoms [1]. It is responsible for a multi-year pandemic and has proven to be a serious infectious agent. This meant that developing a vaccine is very crucial, and therefore a significant number of researchers had focused on the development of vaccines and making them undergo clinical trials. Although approved vaccines had minimal to no immediate adverse effects, the long-term effects were still unclear as they require a longer time to appear. Several mild to moderate adverse effects were reported, including cutaneous changes such as pyoderma gangrenosum [2]. Pyoderma gangrenosum is an uncommon ulcerative auto-inflammatory dermatosis [3], a condition in which non-infectious, non-contagious and large painful ulcers develop on the skin [4]. In this case, we present a 36-year-old adult, otherwise healthy, who developed pyoderma gangrenosum following the first dose of Sinopharm BBIBP COVID-19 vaccine.

## Case presentation

A 36-year-old male was admitted to the hospital after the progression of pyoderma gangrenosum on the lateral aspect of his left arm (deltoid muscle region), which developed eight months ago. The patient received his first Sinopharm COVID-19 vaccine shot on August 18, 2021 on his left arm. He developed redness on his left arm a few hours following the vaccine shot. After four days, the lesion had begun to form, which caused concern to the patient. On that day, he went to a hospital where the initial examination did not cause great concern to the doctors and he was reassured. The patient decided to take Amoxicillin and some painkillers without a prescription. For the next eight months, the patient did not follow up with any medical institution until he noticed a significant progression. The patient stated the progression was an increase in the depth of the lesion and circumference. He then decided to seek medical care and was admitted into the Internal Medicine department.

**Figure 1:**
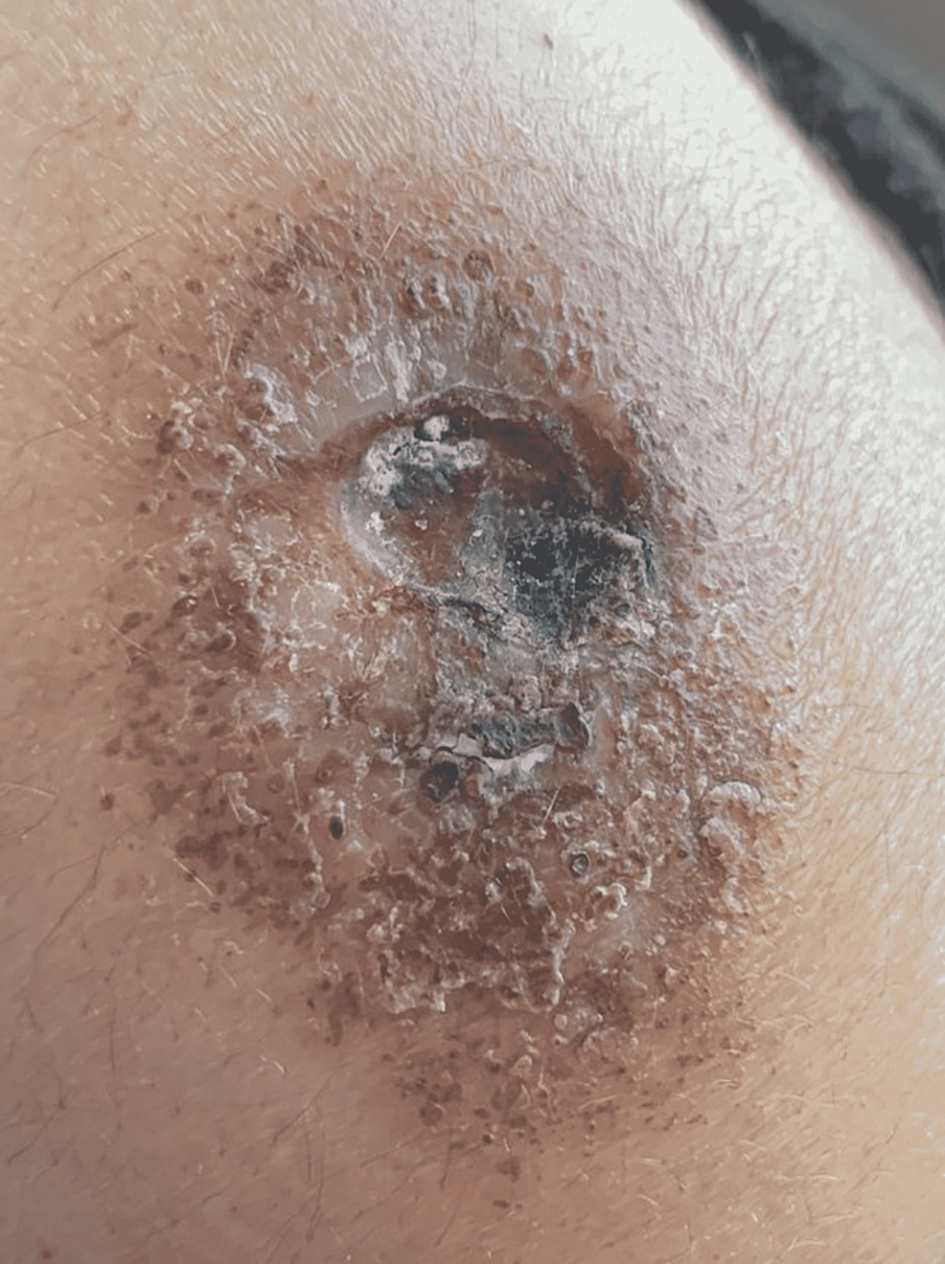
Pyoderma gangrenosum (dated March 10, 2022, eight months after the COVID-19 vaccination): a large ulcer with surrounding erythema and irregular base and border.

**Figure 2:**
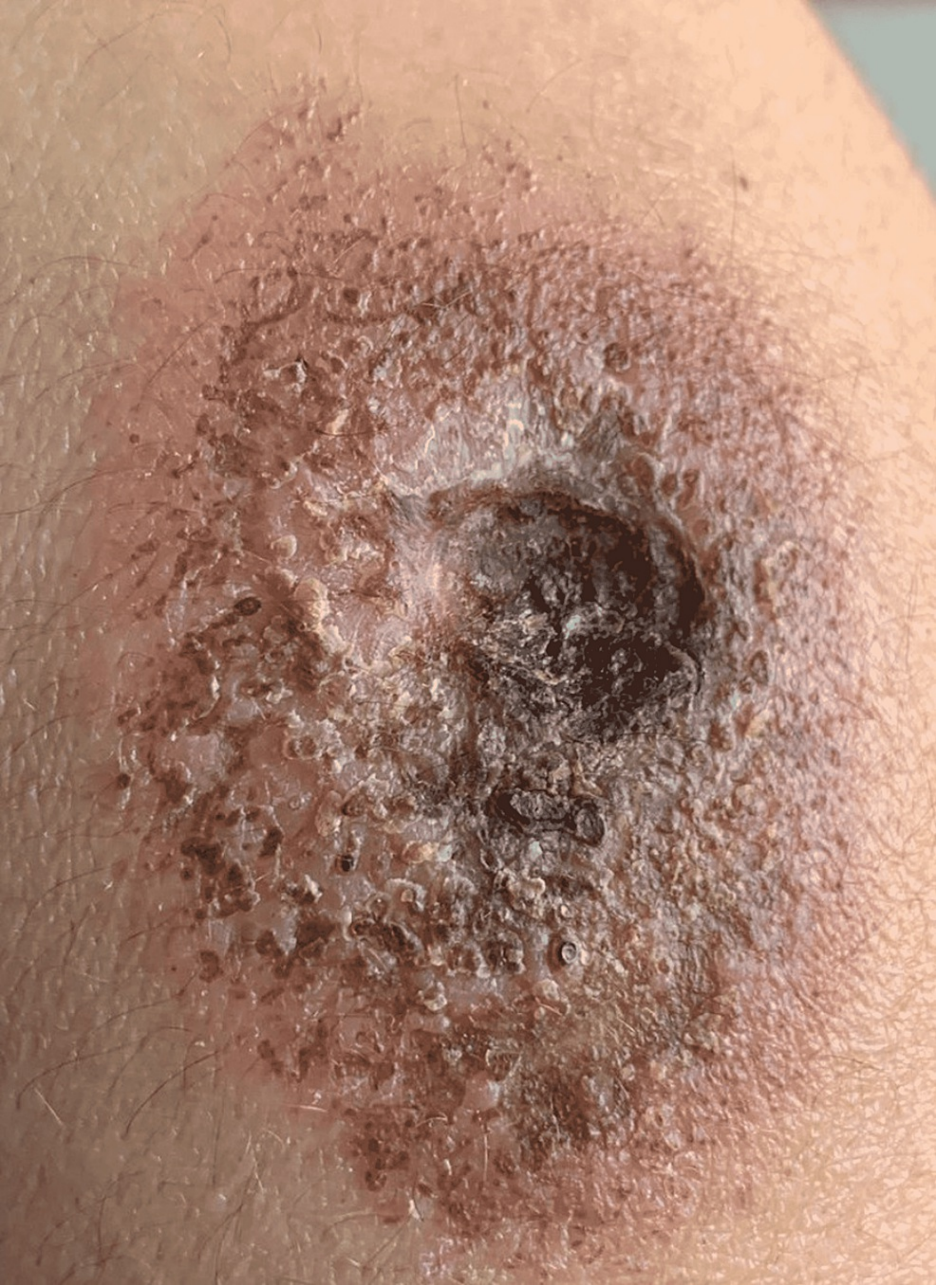
Pyoderma gangrenosum (dated March 14, 2022): the image demonstrates the progression of the lesion despite treatment.

Figure 1 shows a large ulcer with surrounding erythema with an irregular base and border and a surface area of 42 cm^2^. The floor of the ulcer is necrotic and a part of the edge is undermined. The surrounding skin is erythematous. The photo was taken at the time of patient admission, eight months after the COVID-19 vaccination. Figure 2 demonstrates the progression of the lesion in four days during his stay at the hospital. Clearly, the lesion expanded with more necrotic tissue in the surrounding skin, with a surface area of 70 cm^2^, indicating no treatment response. Table 1 provides a timeline of the events.

**Table 1:** A timeline of the events.

Date	Event
August 18, 2021	Frist vaccine shot
August 18, 2021 to August 21, 2021	Redness at the site of vaccine shot
August 22, 2021	Lesion begun to form at the site of vaccine shot. The patient went to a medical institution and was reassured.
August 22, 2021 to March 8, 2022	The lesion had been slowly progressing.
March 9, 2022	The patient noticed the lesion becoming deeper, so he decided to seek medical care.
March 10, 2022	The patient was admitted into the hospital. Figure 1 was taken at the time of patient admission, eight months after the COVID-19 vaccination.
March 14, 2022	Figure 2 demonstrates the progression of the lesion in 4 days during his stay at the hospital

The patient's associated symptoms were headache, blurred vision, palpitation, fatigue on exertion, documented fever, chills and productive cough with yellow sputum. These symptoms started three days prior to admission. He also reported multiple episodes of epistaxis; each episode had a 10-15 minutes’ duration. He stated no unprotected sexual behavior, recent travel, or exposure to any animals, insects or tick bites.

**Figure 3:**
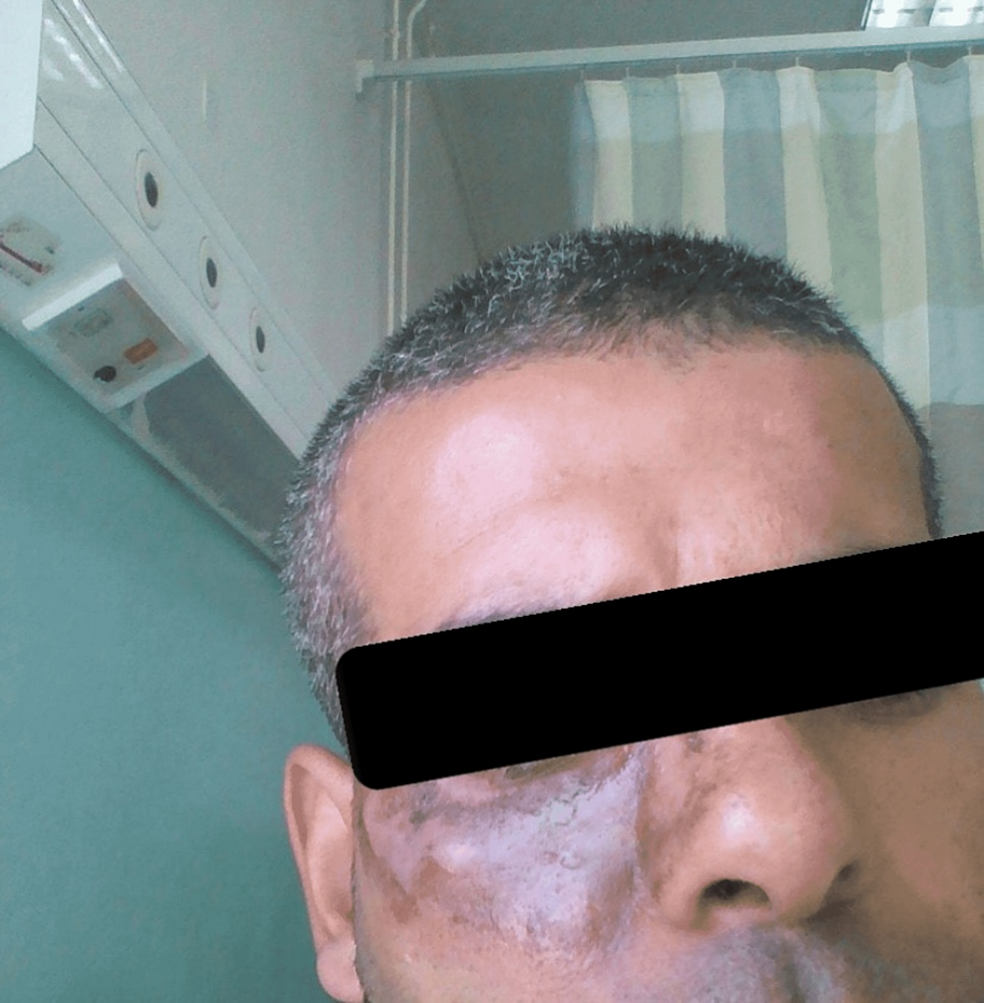
Abnormal skin lesions on the patient's face, eight years prior to vaccination.

His past medical history revealed that he is hypertensive, non-compliant with medication, a smoker and has had leukopenia for an unknown reason. The patient mentioned that he had several abnormal skin lesions in the past on his face that were similar to the newly developed lesion, as shown in Figure 3. Unfortunately, the previous diagnosis and management are unknown due to the lack of access to the patient's medical records. At present, the lesion does not appear on his face. It is worth mentioning that he has no history of malignancy or autoimmune diseases.

In the hospital, a physical examination of the patient revealed that he was conscious, alert, and oriented with normal vital signs. An indurated ulcer on his left arm was detected and an erythematous area surrounded it. Skin pigmentations were identified in the lower limb. No abnormalities were detected in the cardiovascular and respiratory systems. On abdominal examination hepatomegaly was detected.

Lab results indicated that the patient has leukopenia with a WBC count of 1.9 x 10^3^/L (normal range is 4.5-11 x 10^9^/L), neutropenia with an absolute neutrophilic count (ANC) of 0.4 x 10^3^/mL (normal range is 1.5-8 x 10^3^/mL) and thrombocytopenia with a platelet count of 147 x 10^3^ (normal range is 15-45 x 10^4^). Prothrombin time is increased (14.7) seconds (normal range is 11-13.5 seconds). The patient's Hb levels, MCV, MCH, Vit B, folate, liver function test (LFT) and kidney function test (KFT) were all normal. antinuclear AB (ANA) is negative; rheumatoid factor is within normal range. ANA and rheumatoid factor tests are significant as pyoderma gangrenosum is known to be associated with autoimmune and inflammatory systemic disorders [5]. Cancer antigen 15-3, cancer antigen 19-9 and cancer antigen 125 were not detected. Biopsy of the ulcer was critical to exclude malignancy. No pathogens were detected after the culture of the pus, which was critical to exclude any infections.

The diagnosis was made following the Delphi consensus [6]. After exclusion of malignancy and infection, histological examination of the ulcer demonstrated neutrophilic infiltration. One major criterion and three minor criteria were fulfilled. The major criterion was the presence of neutrophilic infiltration on histological examination. The three minor criteria were the exclusion of infection, cribriform scars at healed ulcer sites and history of papule, pustule or vesicle rapidly ulcerating.

Once the patient was admitted to the hospital, he was prescribed Cilastatin/Imipenem (500g IV) and later that day, he was given Cyclosporine (100mg oral). Methylprednisolone (1g IV) was added to the next day's treatment regimen. All three medications were continued to be given for a week. Despite the medications, the lesion did not seem to regress. The patient was discharged and was given follow-up appointments with the dermatology and hematology departments of the hospital after two weeks, as there was no improvement.

## Discussion

This case report discusses an adult who developed pyoderma gangrenosum after the first shot of the BBIBP-CorV COVID-19 vaccine, known as the Sinopharm vaccine. The patient has a past medical history of a similar skin lesion that was present on his face. Hence, the vaccine shot induced a strong response, which may have triggered the reappearance of the pyoderma gangrenosum. Therefore, we suggest a hypothesis that the COVID-19 viral antigen might be an immunological trigger for pyoderma gangrenosum.

In Clark and Williams's study [‎2], a case report shows a 73-year-old woman who developed a pyoderma gangrenosum lesion after receiving Tozinameran (BNT162b2) COVID-19 vaccine. The lesion began to develop after the second shot on her left lower leg which was the site of a previous lesion. Our case differs from the case report in Clark and Williams's study [‎2] in several ways. In our case, the lesion appeared following the first shot of the vaccine. Also, the lesion developed on the site of the vaccine shot which was his left arm not the site of a previous lesion.

Regarding similarities between ours and Clark and Williams's study [‎2], both patients experienced previous lesions. Furthermore, both patients began to develop cutaneous symptoms such as redness and pain a few hours after the vaccine; however, the ulcer development started shortly after the vaccine shot. Both patients also showed no response to medications.

Regarding the mentioned case in Clark and Williams's study [‎2], no tests were reported to exclude other causes of the lesion such as autoimmune causes, infection and malignancy. In our case report, numerous medical tests were done. ANA and rheumatoid factor tests were done to exclude autoimmune diseases. Also, cancer antigen 15-3, cancer antigen 19-9 and cancer antigen 125 were not detected, excluding any malignancy in the body. Furthermore, a biopsy was taken from the lesion to exclude any malignancy in the lesion itself. A culture of the pus was also done to exclude any infections.

The pathogenesis of pyoderma gangrenosum is unclear. However, it is assumed to involve both the innate and adaptive immune systems. It is associated with a genetic factor and neutrophilic abnormalities. The disease is not infectious or communicable. Furthermore, it is frequently linked to different autoimmune disorders [‎3].

A study of an inflammatory gene expression in pathological and non-pathological skin of patients with pyoderma gangrenosum showed that a variety of inflammatory mediators had been increasingly secreted in pyoderma gangrenosum lesions, such as IL-23, IL-1α, IL-1β, IL-6, IL-8, IL-12, and tumor necrosis factor (TNF)-alpha [3,7]. Clinical investigations have revealed that the vaccines trigger human antibody and Th1 cell response, which increases blood levels of IL-2, TNF-α and interferon (IFN)-gamma as a response to COVID-19 viral antigen [8,9]. TNF-α mainly contributes to the secretion and activation of IL-6. This will lead to the binding of IL-6 to the α-interleukin-6 receptor (α -IL-6R), which will promote signal transduction [10].

As a result, this complex will induce the dimerization of the β-receptor gp13; which will cause signal transduction by activating JAK/STAT kinase pathway [10]. This way, the JAK/STAT pathway has a significant role in the inflammatory response to COVID-19 antigen. On the other hand, recent reports and a case-based study suggested that the activation of JAK/STAT pathway plays a key role in the pathophysiology of pyoderma gangrenosum [11].

This proves that the immune response to COVID-19 antigen is a possible trigger for pyoderma gangrenosum, especially in people who have a history of pyoderma gangrenosum or other inflammatory and autoimmune disorders.

Literature review

Multiple skin reactions following SARS-CoV-2 vaccination have been found. These abnormal findings occur either at the injection sites or in other parts of the body. Many of these findings are due to hypersensitivity reactions or autoimmune-mediated pathologies [12]. Some of the cutaneous side effects include urticaria, erythema, delayed inflammatory reactions to dermal fillers, lichen planus, varicella-zoster, herpes simplex, pityriasis rosea and purpura [13].

All the cases reported in the literature regarding pyoderma gangrenosum after SARS-CoV-2 vaccination are summarized in Table 2. There are two cases of reported pyoderma gangrenosum after SARS-CoV-2 vaccination, both occurring after mRNA type (age ranged from 23 to 73 years, one man and one woman), while this case is after the inactivated type. These cases have been reported in countries on different continents, including Asia and North America.

**Table 2:** Cases reported regarding pyoderma gangrenosum after SARS-CoV-2 vaccination

Sex/ Age	Country	Type of Vaccine	Time of onset after vaccination	Past medical history of pyoderma	Chronic illness	Reference
M / 23	KSA	mRNA	24 hours	no	-	[14]
F / 73	USA	mRNA	2 weeks	yes	-	[2]

## Conclusions

Pyoderma gangrenosum might be triggered by COVID-19 vaccinations, particularly in patients who had previously experienced similar lesions in different body parts. Therefore, patients must not neglect cutaneous lesions following COVID-19 vaccinations. Also, clinicians should enquire about the past medical history prior to vaccination to avoid vaccine complications. Furthermore, we propose a national database for the COVID-19 vaccine-related side effects, which should be available to all medical institutions for increased awareness and to report new side effects.
